# Weighted burden analysis of rare coding variants in 470,000 exome-sequenced UK Biobank participants characterises effects on hyperlipidaemia risk

**DOI:** 10.1038/s10038-024-01235-8

**Published:** 2024-03-07

**Authors:** David Curtis

**Affiliations:** UCL Genetics Institute, University College London, London, UK

**Keywords:** Risk factors, Metabolic disorders

## Abstract

A previous study of 200,000 exome-sequenced UK Biobank participants investigating the association between rare coding variants and hyperlipidaemia had implicated four genes, *LDLR*, *PCSK9*, *APOC3* and *IFITM5*, at exome-wide significance. In addition, a further 43 protein-coding genes were significant with an uncorrected *p* value of <0.001. Exome sequence data has become available for a further 270,000 participants and weighted burden analysis to test for association with hyperlipidaemia was carried out in this sample for the 47 genes highlighted by the previous study. There was no evidence to implicate *IFITM5* but *LDLR*, *PCSK9*, *APOC3*, *ANGPTL3*, *ABCG5* and *NPC1L1* were all statistically significant after correction for multiple testing. These six genes were also all exome-wide significant in the combined sample of 470,000 participants. Variants impairing function of *LDLR* and *ABCG5* were associated with increased risk whereas variants in the other genes were protective. Variant categories associated with large effect sizes are cumulatively very rare and the main benefit of this kind of study seems to be to throw light on the molecular mechanisms impacting hyperlipidaemia risk, hopefully supporting attempts to develop improved therapies.

## Introduction

A previous weighted burden analysis of rare coding variants observed in 200,000 exome-sequenced UK Biobank participants implicated four protein-coding genes as being involved in risk of hyperlipidaemia at exome-wide significance: *LDLR*, *PCSK9*, *ANGPTL3* and *IFITM5* [1]. The report of that study also reviewed the broader contribution of genetic variation to hyperlipidaemia risk and that discussion will not be repeated here. For all of these four genes except *LDLR*, rare variants predicted to impair gene functioning were associated with lower risk of hyperlipidaemia. It was noted that overall 55 genes, of which 47 were protein-coding, had uncorrected *p* values significant at *p* < 0.001 whereas only 23 would be expected by chance and a number of these genes seemed to be plausible biological candidates. Exome sequence data for a full set of 470,000 participants has now been released and a follow-up study was carried out in order to determine if any of these 47 genes of interest demonstrated evidence for association in the 270,000 newly available samples. For such genes, further analyses were performed in the whole sample to investigate the overall evidence for association and the contributions from different types of variant.

Most of this exome sequence data has also been used in two previous studies which tested for gene-wise associations with very large numbers of phenotypes, including some hyperlipidaemia-related phenotypes [2, 3]. It was recognised that results from the current investigation would need to be considered in this context of these studies.

## Methods

In order to maintain compatibility with the previous study, the same methods were used for the genetic analyses and for phenotype definition. The description is repeated here for convenience.

### UK Biobank dataset

UK Biobank participants are volunteers intended to be broadly representative of the UK population and are not selected on the basis of having any health condition. UK Biobank had obtained ethics approval from the North West Multi-centre Research Ethics Committee which covers the UK (approval number: 11/NW/0382) and had obtained informed consent from all participants. The UK Biobank approved an application for use of the data (ID 51119) and ethics approval for the analyses was obtained from the UCL Research Ethics Committee (11527/001). The UK Biobank Research Analysis Platform was used to access the Final Release Population level exome OQFE variants in PLINK format for 469,818 exomes which had been produced at the Regeneron Genetics Center using the protocols described here: https://dnanexus.gitbook.io/uk-biobank-rap/science-corner/whole-exome-sequencing-oqfe-protocol/protocol-for-processing-ukb-whole-exome-sequencing-data-sets [3]. All variants were then annotated using the standard software packages VEP, PolyPhen and SIFT [4–6]. To obtain population principal components reflecting ancestry, version 2.0 of *plink* (https://www.cog-genomics.org/plink/2.0/) was run with the options *--maf 0.1 --pca 20 approx* [7, 8].

### Hyperlipidaemia phenotype

The hyperlipidaemia phenotype was determined in the same way as previously from four sources in the dataset: self-reported high cholesterol; reporting taking cholesterol lowering medication; reporting taking a named statin; having an ICD10 diagnosis for hyperlipidaemia in hospital records or as a cause of death [9]. Participants in any of these categories were deemed to be cases with hyperlipidaemia while all other subjects were taken to be controls. As previously described, the UK Biobank sample does contain some subjects who are related to each other [10]. These subjects were not excluded as including them is not theoretically expected to cause major difficulties for the methods of analysis used and using them in previous similar analyses of this dataset had proved unproblematic. When carrying out the original study a deliberate decision had been made to attempt to identify clinically defined cases of hyperlipidaemia without including information on measured lipid levels in the blood, since these levels would likely be influenced by medication. In the primary analyses to implicate specific genes, attention was restricted to participants not included in the earlier study, consisting of 62,066 cases and 207,216 controls. For the subsequent analyses using the whole sample there were 106,091 cases and 363,674 controls.

### Variant weighting

The SCOREASSOC program was used to carry out a weighted burden analysis to test whether, in each gene, sequence variants which were rarer and/or predicted to have more severe functional effects occurred more commonly in cases than controls [11–13]. Attention was restricted to rare variants with minor allele frequency (MAF) ≤0.01 in cases or controls or both. As previously described in detail, variants were weighted by overall MAF so that variants with MAF = 0.01 were given a weight of 1 while very rare variants with MAF close to zero were given a weight of 10. This is done by taking the weight to be a parabolic function of MAF passing through the points (0, 10) and (0.01, 1). Variants were also weighted according to their functional annotation using the GENEVARASSOC program, which was used to generate the input files for weighted burden analysis by SCOREASSOC. Variants predicted to cause complete loss of function (LOF) of the gene were assigned a weight of 100. Nonsynonymous variants were assigned a weight of 5 but if PolyPhen annotated them as possibly or probably damaging then 5 or 10 was added to this and if SIFT annotated them as deleterious then 20 was added. The full set of weights and categories is displayed in Table 1 of the previous study [1]. This means that each variant is assigned a weight according to its MAF and a weight according to its functional annotation. As described previously, the weight due to MAF and the weight due to functional annotation were multiplied together to provide an overall weight for each variant. Variants were excluded if there were more than 10% of genotypes missing in the controls and cases or if the heterozygote count was smaller than both homozygote counts in controls and cases. If a subject was not genotyped for a variant then they were assigned the subject-wise average score for that variant. For each subject a gene-wise weighted burden score was derived as the sum of the variant-wise weights, each multiplied by the number of alleles of the variant which the given subject possessed.

**Table 1:** Genes with absolute value of SLP exceeding 3 (equivalent to *p* < 0.001) for association with hypertension in previous study showing the SLP obtained in the new sample

Symbol	SLP in original sample	Name	SLP in new sample	SLP in combined sample
*LDLR*	50.08	Low Density Lipoprotein Receptor	87.28	156.81
*G6PC*	5.55	Glucose-6-Phosphatase Catalytic Subunit	0.67	
*SULT1E1*	4.63	Sulfotransferase Family 1E Member 1	0.57	
*SLC35G1*	4.38	Solute Carrier Family 35 Member G1	−0.06	
*PLA2G5*	4.15	Phospholipase A2 Group V	1.05	
*CMTM7*	3.99	CKLF Like MARVEL Transmembrane Domain Containing 7	−0.34	
*DEFB131A*	3.66	Defensin Beta 131A	0.43	
*OTULIN*	3.62	OTU Deubiquitinase With Linear Linkage Specificity	−1.10	
*FAM122C*	3.51	Family With Sequence Similarity 122C	0.36	
*CMIP*	3.48	C-Maf Inducing Protein	0.02	
*EIF4B*	3.45	Eukaryotic Translation Initiation Factor 4B	0.52	
*PPP2R3B*	3.41	Protein Phosphatase 2 Regulatory Subunit B“Beta	0.17	
*HNRNPAB*	3.38	Heterogeneous Nuclear Ribonucleoprotein A/B	−0.30	
*PREB*	3.37	Prolactin Regulatory Element Binding	0.93	
*PEX12*	3.36	Peroxisomal Biogenesis Factor 12	−0.12	
*FAM167A*	3.35	Family With Sequence Similarity 167 Member A	0.84	
*ABCG5*	3.31	ATP Binding Cassette Subfamily G Member 5	5.08	6.95
*ABCD1*	3.26	ATP Binding Cassette Subfamily D Member 1	0.18	
*PRAF2*	3.21	PRA1 Domain Family Member 2	−0.45	
*CTHRC1*	3.16	Collagen Triple Helix Repeat Containing 1	−0.33	
*SLC25A37*	3.14	Solute Carrier Family 25 Member 37	0.26	
*CT62*	3.11	Cancer/Testis Associated 62	0.41	
*L1TD1*	3.09	LINE1 Type Transposase Domain Containing 1	0.02	
*PIK3R6*	3.09	Phosphoinositide-3-Kinase Regulatory Subunit 6	1.16	
*FOXO3B*	3.08	Forkhead Box O3B	−0.24	
*FAM47A*	3.06	Family With Sequence Similarity 47 Member A	0.19	
*GCK*	3.04	Glucokinase	1.42	
*MAPKAPK2*	3.02	MAPK Activated Protein Kinase 2	−0.21	
*PCSK9*	−10.42	Proprotein Convertase Subtilisin/Kexin Type 9	−29.08	−43.57
*IFITM5*	−5.86	Interferon Induced Transmembrane Protein 5	0.44	
*ANGPTL3*	−5.67	Angiopoietin Like 3	−7.48	−12.68
*APOC3*	−4.89	Apolipoprotein C3	−11.01	−13.19
*PPP1R3G*	−4.25	Protein Phosphatase 1 Regulatory Subunit 3G	−0.67	
*TBC1D8*	−3.93	TBC1 Domain Family Member 8	−0.90	
*CTXN2*	−3.91	Cortexin 2	−0.64	
*NPC1L1*	−3.7	NPC1 Like Intracellular Cholesterol Transporter 1	−3.42	−7.60
*ANGPTL4*	−3.66	Angiopoietin Like 4	−1.95	−4.79
*SNX17*	−3.62	Sorting Nexin 17	−0.73	
*SV2B*	−3.29	Synaptic Vesicle Glycoprotein 2B	0.23	
*ITM2B*	−3.23	Integral Membrane Protein 2B	0.40	
*UBR4*	−3.23	Ubiquitin Protein Ligase E3 Component N-Recognin 4	0.82	
*TXNL4A*	−3.22	Thioredoxin Like 4A	0.11	
*TTR*	−3.16	Transthyretin	−0.38	
*GFPT1*	−3.1	Glutamine--Fructose-6-Phosphate Transaminase 1	0.09	
*APPBP2*	−3.08	Amyloid Beta Precursor Protein Binding Protein 2	0.29	
*CRYZL1*	−3.06	Crystallin Zeta Like 1	−0.70	
*HLA-A*	3.01	Major Histocompatibility Complex, Class I, A	0.46	

SLPs significant after correction for multiple testing are shown in bold. For all genes achieving an absolute value of SLP exceeding 2.97 and for *GUCY1B1*, the SLP obtained for combined sample is also shown

### Logistic regression analysis

Analyses were restricted to the 47 protein-coding genes significant at *p* < 0.001 in the previous study. For each gene, logistic regression analysis was carried out with hyperlipidaemia as the dependent variable including the first 20 population principal components and sex as covariates and a likelihood ratio test was performed comparing the likelihoods of the models with and without the gene-wise burden score. This is a test for association between the gene-wise burden score and caseness and the statistical significance was summarised as a signed log *p* value (SLP), which is the log base 10 of the *p* value given a positive sign if the score is higher in cases and negative if it is higher in controls. Since only 47 genes were analysed, after correcting for multiple testing a gene could be declared statistically significant if it achieved an SLP with absolute value greater than -log10(0.05/47) = 2.97 using the new samples.

### Follow-up analyses

Follow-up analyses were performed on all genes individually achieving this significance level and also *ANGPTL4*. For this subset of genes the weighted burden analysis described above was carried out using the whole sample of 106,091 cases and 363,674 controls. Additionally, for each subject a count was obtained of the number of variants they carried falling into particular broad annotation categories, such as LOF, protein altering, etc. The full list of these categories is shown in Supplementary Table 1. These counts were entered into a multiple logistic regression analysis with hyperlipidaemia as the dependent variable and again including sex and 20 principal components as covariates in order to elucidate the contribution of different types of variant to the overall evidence for association. The odds ratios (ORs) associated with each category were estimated along with their standard errors and the Wald statistic was used to obtain a *p* value. This *p* value was converted to an SLP, again with the sign being positive if the OR was greater than 1, indicating that variants in that category tended to increase risk.

### Software

Data manipulation and statistical analyses were performed using GENEVARASSOC, SCOREASSOC and R [12–14].

## Results

Table 1 shows the results of the primary analysis. Three of the four protein-coding genes which reach exome-wide significance in the earlier study show convincing evidence of association with hyperlipidaemia, *LDLR*, *PCSK9* and *ANGPTL3*, with SLPs of 87.28, −29.08 and −7.48 respectively. However the other gene, *IFITM5*, shows no evidence of association, with SLP of only 0.44, so it seems reasonable to conclude that the original results for this gene represented a type 1 error. Of the remaining genes which were originally significant at *p* < 0.001, most show no evidence of association in this new sample and can be dismissed as chance findings. However *ABCG5*, *APOC3* and *NPC1L1* all produce SLPs which are statistically significant after correcting for testing 47 genes, with values of 5.08, −11.01 and −3.42. These six genes were carried forward for secondary analyses along with *ANGPTL4* which was considered to be of interest because of its similarity to *ANGPTL3*, even though it achieved SLPs of only −3.66 in the first sample and −1.95 in the second.

The original study considered 22,642 genes, meaning that for a gene-wise result to be considered exome-wide significant the magnitude of the SLP obtained should exceed -log10(0.05/22642) = 5.66. For the seven genes carried forward, the results of weighted burden analysis in the entire sample of 106,091 cases and 363,674 controls are also shown in Table 1 and it can be seen that all six of the genes which produced results which were statistically significant after multiple testing in the second sample also produce results which would be regarded as exome-wide significant in the full sample. However the SLP for *ANGPTL4* in the combined sample is only −4.79.

In order to gain insights into the effects of different categories of variant within these seven genes of interest, counts for variants of each category in each subject were entered into multiple logistic regression analysis along with sex and 20 principal components as covariates. These results are shown in Tables 2–4 and are summarised briefly as follows.

**Table 2:** Results from logistic regression analysis showing the contribution different categories of variant within each gene make to risk of hyperlipidaemia

A							
Results for *LDLR*.							
Variant category	Number of separate variants	Total count in controls	Mean count in controls	Total count in cases	Mean count in cases	OR (95% confidence interval)	SLP
Intronic, etc	1116	62789	0.172651	18763	0.176854	1.03 (1.01–1.04)	2.80
5 prime UTR	46	172	0.000473	44	0.000415	0.89 (0.63–1.26)	−0.30
Synonymous	283	5947	0.016353	1639	0.015449	0.93 (0.88–0.98)	−2.02
Splice region	81	15506	0.042638	4022	0.037911	0.82 (0.79–0.86)	−19.81
3 prime UTR	27	1248	0.003433	342	0.003225	0.95 (0.84–1.08)	−0.39
Protein altering	624	11496	0.031611	4287	0.040409	1.12 (1.06–1.19)	4.43
Indel, etc	6	2	0.000005	17	0.000160	25.66 (5.67–116.17)	8.96
LOF	44	16	0.000044	97	0.000914	23.48 (13.63–40.46)	30.39
SIFT deleterious	329	1736	0.004774	1148	0.010821	1.44 (1.26–1.65)	7.15
PolyPhen possibly damaging	123	964	0.002651	472	0.004449	1.27 (1.10–1.46)	3.16
PolyPhen probably damaging	197	1299	0.003572	898	0.008465	1.61 (1.39–1.86)	9.80
**B**							
**Results for** ***ABCG5***.							
Intronic, etc	1370	30573	0.084068	9093	0.085713	1.01 (0.99–1.03)	0.49
5 prime UTR	77	517	0.001422	158	0.001489	0.99 (0.83–1.19)	−0.03
Synonymous	222	3828	0.010526	1218	0.011481	1.04 (0.97–1.11)	0.52
Splice region	74	674	0.001854	271	0.002555	1.44 (1.24–1.66)	6.20
3 prime UTR	7	18	0.000049	2	0.000019	0.35 (0.08–1.56)	−0.55
Protein altering	520	17153	0.047166	5476	0.051619	1.05 (1.01–1.09)	2.07
Indel, etc	1	0	0.000000	2	0.000019		1.29
LOF	58	368	0.001012	128	0.001207	1.20 (0.98–1.48)	1.12
SIFT deleterious	290	5381	0.014797	1831	0.017259	1.08 (0.96–1.23)	0.69
PolyPhen possibly damaging	96	2104	0.005785	694	0.006542	0.98 (0.85–1.13)	−0.10
PolyPhen probably damaging	161	2321	0.006382	813	0.007663	1.05 (0.92–1.21)	0.33

Odds ratios for each category are estimated including principal components and sex as covariates

**Table 3:** Results of variant category analysis for *PCSK9, NPC1L1* and *APOC3*

A							
Results for *PCSK9*.							
Variant category	Number of separate variants	Total count in controls	Mean count in controls	Total count in cases	Mean count in cases	OR (95% confidence interval)	SLP
Intronic, etc	695	13345	0.036694	4024	0.037926	1.03 (1.00–1.07)	1.21
5 prime UTR	48	657	0.001807	186	0.001753	0.98 (0.83–1.16)	−0.09
Synonymous	230	16789	0.046165	4999	0.047120	0.99 (0.96–1.03)	−0.15
Splice region	55	698	0.001919	223	0.002102	1.06 (0.91–1.24)	0.36
3 prime UTR	40	5701	0.015676	1719	0.016206	1.02 (0.97–1.08)	0.34
Protein altering	537	7905	0.021737	1891	0.017824	0.92 (0.86–0.98)	−1.93
Indel, etc	10	2620	0.007205	758	0.007145	1.00 (0.92–1.08)	−0.03
LOF	72	862	0.002370	94	0.000886	0.39 (0.31–0.48)	−17.41
SIFT deleterious	244	3354	0.009223	655	0.006174	0.69 (0.59–0.81)	−5.46
PolyPhen possibly damaging	70	616	0.001694	177	0.001668	1.18 (0.98–1.43)	1.12
PolyPhen probably damaging	157	2408	0.006621	475	0.004477	1.01 (0.85–1.21)	0.05
**B**							
**Results for** ***NPC1L1***.							
Intronic, etc	1197	30977	0.085178	9035	0.085162	1.00 (0.97–1.02)	−0.13
5 prime UTR	24	853	0.002346	275	0.002592	1.14 (0.99–1.31)	1.19
Synonymous	442	11147	0.030651	3218	0.030333	0.98 (0.94–1.02)	−0.54
Splice region	74	605	0.001664	164	0.001546	0.94 (0.79–1.13)	−0.30
3 prime UTR	31	954	0.002624	282	0.002659	0.96 (0.84–1.10)	−0.25
Protein altering	926	28236	0.077642	7764	0.073184	0.98 (0.96–1.00)	−0.94
Indel, etc	13	43	0.000118	16	0.000151	1.31 (0.72–2.38)	0.44
LOF	100	661	0.001818	123	0.001160	0.64 (0.53–0.78)	−5.20
SIFT deleterious	507	8949	0.024607	2366	0.022302	0.89 (0.80–0.98)	−1.76
PolyPhen possibly damaging	143	5970	0.016416	1532	0.014441	1.00 (0.90–1.11)	−0.01
PolyPhen probably damaging	325	2798	0.007694	817	0.007701	1.15 (1.01–1.30)	1.52
**C**							
**Results for** ***APOC3***.							
Intronic, etc	134	10194	0.028031	2817	0.026555	0.95 (0.91–1.01)	−1.13
5 prime UTR	2	1	0.000003	1	0.000009	3.51 (0.20–62.29)	0.4
Synonymous	31	207	0.000569	48	0.000452	0.81 (0.59–1.12)	−0.7
Splice region	11	119	0.000327	37	0.000349	1.06 (0.72–1.55)	0.11
3 prime UTR	33	199	0.000547	56	0.000528	1.01 (0.75–1.37)	0.03
Protein altering	69	568	0.001562	136	0.001282	1.06 (0.77–1.45)	0.13
Indel, etc	2	48	0.000132	8	0.000075	0.58 (0.27–1.25)	−0.81
LOF	10	1993	0.005480	402	0.003789	0.68 (0.61–0.76)	−11.22
SIFT deleterious	30	416	0.001144	89	0.000839	0.47 (0.22–1.02)	−1.29
PolyPhen possibly damaging	14	40	0.000110	18	0.000170	2.77 (1.15–6.68)	1.69
PolyPhen probably damaging	10	325	0.000894	66	0.000622	1.35 (0.63–2.92)	0.37

**Table 4:** Results of variant category analysis for *ANGPTL3* and *ANGPTL4*

A							
Results for *ANGPTL3*.							
Variant category	Number of separate variants	Total count in controls	Mean count in controls	Total count in cases	Mean count in cases	OR (95% confidence interval)	SLP
Intronic, etc	347	8024	0.022064	2585	0.024370	1.02 (0.96–1.08)	0.29
5 prime UTR	25	3604	0.009909	1104	0.010408	0.99 (0.87–1.14)	−0.04
Synonymous	103	4397	0.012091	1322	0.012461	0.96 (0.86–1.08)	−0.28
Splice region	22	803	0.002208	161	0.001518	0.69 (0.58–0.82)	−4.70
3 prime UTR	34	2019	0.005550	560	0.005280	0.97 (0.88–1.06)	−0.32
Protein altering	265	10476	0.028806	2945	0.027759	0.94 (0.85–1.03)	−0.77
Indel, etc	6	93	0.000256	20	0.000189	0.69 (0.42–1.15)	−0.84
LOF	56	835	0.002296	143	0.001348	0.59 (0.49–0.70)	−8.36
SIFT deleterious	137	6886	0.018935	1930	0.018192	0.97 (0.86–1.08)	−0.26
PolyPhen possibly damaging	41	7682	0.021123	2175	0.020501	1.07 (0.94–1.23)	0.54
PolyPhen probably damaging	69	708	0.001947	193	0.001819	1.00 (0.81–1.24)	0.01
**B**							
**Results for** ***ANGPTL4***.							
Intronic, etc	354	13934	0.038315	4030	0.037985	1.00 (0.96–1.03)	−0.04
5 prime UTR	38	1469	0.004039	406	0.003827	0.95 (0.83–1.08)	−0.38
Synonymous	146	7038	0.019353	2036	0.019191	1.03 (0.97–1.09)	0.47
Splice region	28	1623	0.004463	482	0.004543	1.01 (0.93–1.09)	0.11
3 prime UTR	41	3410	0.009375	939	0.008852	0.93 (0.84–1.03)	−0.77
Protein altering	321	9388	0.025814	2634	0.024828	0.97 (0.90–1.05)	−0.34
Indel, etc	1	2	0.000005	0	0.000000		0
LOF	43	528	0.001452	121	0.001141	0.78 (0.64–0.96)	−1.84
SIFT deleterious	174	3928	0.010801	985	0.009284	0.89 (0.75–1.05)	−0.83
PolyPhen possibly damaging	52	2618	0.007199	783	0.007380	1.08 (0.97–1.21)	0.82
PolyPhen probably damaging	113	3211	0.008829	808	0.007616	1.01 (0.84–1.22)	0.04

Variants in LDLR (SLP = 156.81) and ABCG5 (SLP = 6.95) increase risk of hyperlipidaemia and results for each variant category are shown in Table 2. Table 2A shows the results for *LDLR* and it can be seen that LOF variants are associated with hyperlipidaemia risk with OR > 20. 113 participants carry a LOF variant and all but 16 of these are cases. Of note, there are also 19 subjects who carry an inframe indel and all but 2 of these are also cases, again yielding an OR over 20 though with a wide confidence interval. Detailed inspection of these results reveals that they are driven by two inframe deletions, 19:11105556ATGG > A (rs121908027) which is carried by 8 participants who are all cases and 19:11116925ACGG > A (rs1221971156) which is carried by 7 participants, 6 of whom are cases. The first of these, rs121908027, is reported to the be most common familial hyperlipidaemia (FH) mutation in Ashkenazi Jews and was found in 35% of FH families in Israel [15]. As well as the large effect of these LOF and indel variants there is statistically significant evidence for an overall small effect on risk of the much commoner variants in the “Protein altering” category (consisting mostly of nonsynonymous variants) with OR of 1.12 and a further modest increase in risk if these are annotated as deleterious by SIFT and/or possibly or probably damaging by PolyPhen, with ORs of 1.44, 1.27 and 1.61. While all these categories of variant are associated with increased risk of hyperlipidaemia, the category “Splice region” is actually associated with reduced risk, with OR of 0.82 and SLP of −19.81. This result is driven by 19:11120527 G > A (rs72658867), which has MAF 0.0126 in controls and 0.0096 in cases and which has previously been reported to lower HDL cholesterol and to be protective against coronary artery disease [16].

Table 2B shows the results for *ABCG5* and it can be seen that although a few hundred participants carry LOF variants these do not appear to have any strong effect on hyperlipidaemia risk with an OR of 1.2 which is not statistically significant. Instead, the signal for this gene seems to be driven largely by the “Splice region category”, with OR of 1.44 and SLP of 6.20. Although there are 74 variants in this category, the result seems to be mainly driven by three variants which are somewhat commoner in cases, 2:43813316 A > C (rs114780578), 2:43822939 G > C (rs370895243) and 2:43825025 A > T (rs201469377). The category “Protein altering” yields an OR of 1.05 and an SLP of 2.07 but there is no suggestion that nonsynonymous variants recognised as more severe by SIFT or PolyPhen are associated with increased risk. This result may be largely driven by 2:43813208 T > C (rs140374206) which had frequency 0.006049 in controls and 0.006246 in cases and which has previously been reported to be associated with raised non-HDL cholesterol and increased risk of gallstones [17].

Variants in *PCSK9* (SLP = −48.57), *NPC1L1* (SLP = −7.60) and *APOC3* (SLP = −13.19) are protective against hyperlipidaemia and their results detailed are shown in Table 3. As can be seen in Table 3A, LOF variants in *PCSK9* reduce hyperlipidaemia risk, with OR of 0.39. On average, protein altering variants in general have a mild effect on lowering risk, with OR of 0.92, but those which are additionally annotated as deleterious by SIFT have a larger effect, with OR 0.69, whereas there is no additional effect associated with being characterised as possibly or probably damaging by PolyPhen.

The results in Table 3B show that the overall signal for *NPC1L1* is mainly due to LOF variants, with OR 0.64 and SLP of −5.20, with possibly some additional contribution from variants annotated as deleterious by SIFT, which have OR 0.89 and SLP −1.76. A similar scenario is seen for *APOC3* in Table 3C, with LOF variants have OR of 0.68 and SLP of −11.22 but with other categories not showing clear evidence of association.

Variants in *ANGPTL3* (SLP = −12.68) and *ANGPTL4* (SLP = −4.79) also appear to be protective against hyperlipidaemia, although the result for *ANGPTL4* is not exome-wide significant. Nevertheless, as the products of both genes modulate the activity of lipoprotein lipase and as inactivating variants in both genes have previously been shown to be associated with hypolipidaemia, it seems appropriate to present the detailed results for both, as shown in Table 4 [18, 19]. For *ANGPTL3* the signal is again mainly due to LOF variants, with OR of 0.59 and SLP of −8.36, but it can be seen that splice region variants also have OR of 0.69 and SLP of −4.70. This latter result is driven by 1:62598067 T > C (rs372257803) which has MAF 0.00100 in controls and 0.00069 in cases and which has been previously reported to be associated with lower non-HLD cholesterol and triglycerides [20]. The results for *ANGPTL4* are not statistically significant after correction for multiple testing but it can be seen that they are consistent with the possibility that LOF variants lower hyperlipidaemia risk modestly, with OR 0.78 and SLP −1.84.

## Discussion

As mentioned above, this dataset has been used for previous two studies testing for association between exome sequence variance with a very large number of phenotypes, which for convenience we can refer to as the Regeneron and AstraZeneca studies [2, 3]. The Regeneron study carried out a variety of single variant and gene-wise burden tests on 3994 health-related traits to produce a total of about 2.3 billion tests, yielding a critical *p* value of 2.18 × 10^−11^ (corresponding to SLP = 10.66)) and reported 8865 significant associations which are presented in their Supplementary Data 2 [3]. This reports significant gene-wise associations of measured cholesterol and/or LDL levels for all seven of the genes implicated in the present study. However the only genes reported to be associated with clinical hyperlipidaemia, as indicated by taking lipid lowering medication or having a hyperlipidaemia diagnosis recorded, were *LDLR*, *PCSK9* and *APOC3*. For the AstraZeneca study, all gene-wise and variant-wise associations with 17,361 binary and 1419 quantitative phenotypes are reported on the AstraZeneca PheWAS Portal at https://azphewas.com/ [2]. This was accessed to find the most significant *p* value for any analysis of each of these genes with the phenotype which on the website is labelled “Union#E78#E78 Disorders of lipoprotein metabolism and other lipidaemias” and Table 5 shows the results obtained compared with those for the current study. This shows that, for every gene, the AstraZeneca results provide less support for association than the present study and in particular both *ABCG5* and *NPC1L1* would be regarded as exome-wide significant in the present study. While, these two genes were both previously shown to influence lipid levels, the current study implicates variants in these genes as impacting the risk of developing clinically relevant hyperlipidaemia, suggesting that such variants could be included when drawing up genetically informed individual risk profiles.

**Table 5:** Comparison of results from current study to those reported for the AstraZeneca study

Gene	SLP for combined sample in current study	SLP for AstraZeneca study
*LDLR*	156.81	70.73
*ABCG5*	6.95	3.94
*PCSK9*	−43.57	−17.12
*NPC1L1*	−7.60	5.21
*APOC3*	−13.19	−12.47
*ANGPTL3*	−12.68	−11.66
*ANGPTL4*	−4.79	−2.08

The results for the AstraZeneca study are displayed as the equivalent SLP for the most significant result reported for that gene with the phenotype “Union#E78#E78 Disorders of lipoprotein metabolism and other lipidaemias”

Of the four genes in which variants impairing function are associated with reduced risk of hyperlipidaemia, two are already established drug targets. The product of *NPC1L1*, which is essential for intestinal sterol absorption, is the molecular target of ezetimibe, a cholesterol absorption inhibitor that lowers blood cholesterol [21]. The product of *ANGPTL3* is the target of evinacumab, a human monoclonal antibody designed to treat hypercholesterolaemia [18, 22]. The therapeutic role of ANGPTL3 inhibition has recently been reviewed [23]. The well-established evidence for *PCSK9* and *APOC3* variants as being protective is fuelling research into developing strategies to find novel ways to antagonise PCSK9 and lower apoC-III [24, 25].

The detailed analyses of variant categories provide insights into the magnitude of effects of different kinds of variant in different genes, along with information about their cumulative frequencies. One feature of note is the heterogeneity of effects between genes. For example, for *LDLR* nonsynonymous variants classified as probably damaging by PolyPhen are more strongly implicated and have a larger effect size than variants classified as deleterious by SIFT, but for *PCSK9* this is not the case and only SIFT deleterious variants have an effect. For *ANGPTL3* neither SIFT nor PolyPhen is helpful for identifying risk-associated variants. For most genes LOF variants have the largest effect size but this is not the case for *ABCG5* in which LOF variants do not clearly have any effect and the signal is instead driven by specific splice region variants. This inconsistency of effects of different methods for weighting across different genes has been noted in a previous study [26]. A total of 43 different methods of predicting the pathogenic effect of nonsynonymous variants were compared across ten different genes associated with common phenotypes, including *LDLR*, *PCSK9* and *ANGPTL3*. These results showed that while SIFT and PolyPhen performed reasonably well for some genes, other methods were better for other genes. However no single prediction method performed consistently well across all genes. Using additional prediction methods would introduce complications around correcting for multiple testing and interpretation of results but it seems that there is no single weighting system which would be optimal for all genes.

Using biobank data to identify genes influencing complex traits contrasts with the strategy of concentrating on individuals and families with extreme phenotypes who may harbour variants with quasi-Mendelian effects [27]. Ascertaining cases from biobank data can yield large sample sizes but with less severe and less well-defined phenotypes. The present study quantifies the effect on clinically relevant hyperlipidaemia risk of naturally occurring variation within the identified genes, along with their cumulative frequencies in a sample broadly representative of the population. The fact that people with severe, early onset cardiovascular disease might have been less likely to survive to be recruited into UK Biobank may mean that the magnitude of effect of some variants on risk is somewhat underestimated, but this does not seem likely to be a major consideration. Variants with large effect sizes are very rare. Around 10,000 participants carry a variant in a category with a moderate effect on risk, i.e. with OR below 0.7 or above 1.4, but it is debatable how relevant such effects would be for quantifying individual risk in the context of personalised medicine. Additionally, we may note that, although formal analyses were not carried out, the individual allele frequencies of these variants would be too low to have an appreciable effect of any common variants which might be in linkage disequilibrium with them, such as could be detected in genome-wide association studies. It seems that the main value of identifying coding variants associated with risk is to clarify the pathophysiological mechanisms influencing risk of hyperlipidaemia in order to support the development of novel therapeutic approaches.

### Supplementary information

Supplementary Table 1

## Data Availability

The raw data is available on application to UK Biobank. Detailed results with variant counts cannot be made available because they might be used for subject identification. Relevant derived variables including principal components and variant annotations will be deposited in UK Biobank. Scripts and software used to carry out the analyses are available at https://github.com/davenomiddlenamecurtis.
