# Supplementary material for: Weighted burden analysis of rare coding variants in 470,000 exome-sequenced UK Biobank participants characterises effects on hyperlipidaemia risk
Source: J Hum Genet. 2024 Mar 7;69(6):255–62. doi: 10.1038/s10038-024-01235-8 (PMC11126377; doi:10.1038/s10038-024-01235-8)
Supplement: Supplementary file 1 — Supplementary Table 1 [file 10038_2024_1235_MOESM1_ESM.docx]

**Weighted burden analysis of rare coding variants in 470,000 exome-sequenced UK Biobank subject characterises effects on hyperlipidaemia risk**

David Curtis

**Supplementary Table 1**

The table shows the broad categories used for variant category specific analyses along with the annotations produced by VEP which were grouped into each category.

| Category | VEP annotation |
| --- | --- |
| Intronic etc. | feature_truncation, regulatory_region_variant, feature_elongation, regulatory_region_amplification, regulatory_region_ablation, TF_binding_site_variant, TFBS_amplification, TFBS_ablation, downstream_gene_variant, upstream_gene_variant, non_coding_transcript_variant, NMD_transcript_variant, intron_variant, non_coding_transcript_exon_variant |
| Five prime UTR | 5_prime_UTR_variant |
| Synonymous | synonymous_variant |
| Splice region | splice_region_variant |
| Three prime UTR | 3_prime_UTR_variant |
| Protein altering | protein_altering_variant, missense_variant |
| Indel etc. | inframe_deletion, inframe_insertion, transcript_amplification |
| LOF | frameshift_variant, stop_gained , transcript_ablation, splice_donor_variant, splice_acceptor_variant |
| SIFT deleterious | deleterious |
| PolyPhen possibly damaging | possibly_damaging |
| PolyPhen probably damaging | probably_damaging |
